# Interactive Effects of Dopamine Baseline Levels and Cycle Phase on Executive Functions: The Role of Progesterone

**DOI:** 10.3389/fnins.2017.00403

**Published:** 2017-07-13

**Authors:** Esmeralda Hidalgo-Lopez, Belinda Pletzer

**Affiliations:** Department of Psychology and Centre for Cognitive Neuroscience, University of Salzburg Salzburg, Austria

**Keywords:** menstrual cycle, estradiol, progesterone, dopamine, executive functions, eye blink rate, working memory, cognitive flexibility

## Abstract

Estradiol and progesterone levels vary along the menstrual cycle and have multiple neuroactive effects, including on the dopaminergic system. Dopamine relates to executive functions in an “inverted U-shaped” manner and its levels are increased by estradiol. Accordingly, dopamine dependent changes in executive functions along the menstrual cycle have been previously studied in the pre-ovulatory phase, when estradiol levels peak. Specifically it has been demonstrated that working memory is enhanced during the pre-ovulatory phase in women with low dopamine baseline levels, but impaired in women with high dopamine baseline levels. However, the role of progesterone, which peaks in the luteal cycle phase, has not been taken into account previously. Therefore, the main goals of the present study were to extend these findings (i) to the luteal cycle phase and (ii) to other executive functions. Furthermore, the usefulness of the eye blink rate (EBR) as an indicator of dopamine baseline levels in menstrual cycle research was explored. 36 naturally cycling women were tested during three cycle phases (menses–low sex hormones; pre-ovulatory–high estradiol; luteal–high progesterone and estradiol). During each session, women performed a verbal N-back task, as measure of working memory, and a single trial version of the Stroop task, as measure of response inhibition and cognitive flexibility. Hormone levels were assessed from saliva samples and spontaneous eye blink rate was recorded during menses. In the N-back task, women were faster during the luteal phase the higher their progesterone levels, irrespective of their dopamine baseline levels. In the Stroop task, we found a dopamine-cycle interaction, which was also driven by the luteal phase and progesterone levels. For women with higher EBR performance decreased during the luteal phase, whereas for women with lower EBR performance improved during the luteal phase. These findings suggest an important role of progesterone in modulating dopamine-cycle interactions. Additionally, we identified the eye blink rate as a non-invasive indicator of baseline dopamine function in menstrual cycle research.

## Introduction

During the past decades, cognitive neuroscience has witnessed an increasing interest in the research of menstrual cycle and sex hormone effects on female brain and cognition. In naturally cycling women, estradiol and progesterone levels fluctuate along the menstrual cycle as a product of complex neuroendocrine interactions. In the early follicular phase (to which we will refer as *menses* phase), both estradiol and progesterone levels are low, whereas the late follicular phase (or *pre-ovulatory* phase), is characterized by a peak in estradiol levels, and the *luteal* phase by high levels of progesterone and medium of estradiol (Fehring et al., 2006; Sacher et al., 2013).

Both estradiol and progesterone are known to interact with several neurotransmitter systems, including norepinephrine, serotonin, acetylcholine and dopamine (DA) (Genazzani et al., 1997; McEwen and Alves, 1999; Mitsushima, 2010; Barth et al., 2015). In particular, estradiol seems to have a special impact on the dopaminergic system. Animal research has demonstrated numerous genomic and non-genomic estradiol effects on functional activity modulating DA. It increases the synthesis, release, reuptake and turnover of DA, affects downstream targets of its receptor and modifies basal firing rates of dopaminergic neurons (Tansey et al., 1983; Becker, 1990, 1999; Bazzett and Becker, 1994; Pasqualini et al., 1995; Bethea et al., 2002). The effect of progesterone on neurotransmitter systems is not as straightforward. While some studies assume that progesterone exerts opposite effects to estradiol (Fernández-Ruiz et al., 1990), others assume similar effects of both hormones (Sánchez et al., 2010) or a modulation of the estrogenic actions by progesterone (Alves et al., 2000; Barbosa-Vargas et al., 2009). Likewise, for the dopaminergic system, some studies reported an effect of progesterone only when primed with estrogen (Dluzen and Ramirez, 1987; Becker and Rudick, 1999), or effects in opposite direction depending on its concentration (Cabrera et al., 1993). Given the multitude of estrogenic effects on the dopaminergic system, these inconsistencies are not so surprising, as progesterone might facilitate some of these actions, while modulating or opposing others. However, along the menstrual cycle, it is very hard to dissociate the effects of progesterone from the effects of estradiol, since both hormones levels are high during the luteal phase.

Related to these neuroactive effects of estradiol and progesterone, cognitive performance and behavior change along the menstrual cycle as these hormones fluctuate. Specifically performance in tasks which reveal greater gender differences are mediated through sex hormone levels (Hampson, 1990; Kimura and Hampson, 1994; Phillips and Silverman, 1997; Andreano and Cahill, 2009). For instance, verbal abilities seem to be improved during pre-ovulatory (Hampson, 1990) or mid-luteal phase (Maki et al., 2002) whereas spatial abilities, like mental rotation, are improved during menses (Hampson, 1990; Hausmann et al., 2000), and multiple memory systems seem to be differently regulated by ovarian hormones (Phillips, 1992; Hussain et al., 2016). Changes have also been observed in higher cognitive functions, such as working memory (Jacobs and D'Esposito, 2011), cognitive control (Hatta and Nagaya, 2009), inhibitory control (Colzato et al., 2010), navigation task strategy (Hussain et al., 2016) or delayed reward selection (Smith et al., 2014) during the pre-ovulatory phase compared to the menses and/or luteal phase.

However, these findings are far from consistent (Maki et al., 2002; Rosenberg and Park, 2002; Schöning et al., 2007; Gasbarri et al., 2008; Mordecai et al., 2008; Solís-Ortiz and Corsi-Cabrera, 2008). Some of the discrepant results may be explained by the lack of methodological standardization regarding the timing of assessment, confirmation of cycle phase apart from self-reports, or use of biological samples, among others (Sacher et al., 2013; Sundström Poromaa and Gingnell, 2014). With regards to higher cognitive functions it has been suggested that these inconsistencies could also arise from the interactions between estradiol and the dopaminergic system (Colzato and Hommel, 2014). The dopaminergic system plays a key role in complex cognitive processes. Among these higher executive functions, “shifting,” “updating,” and “inhibition” (Miyake et al., 2000) can be distinguished. Since DA modulates higher cognitive functions in an inverted u-shaped manner, either insufficient or excessive levels of this neurotransmitter are related to a worse performance. The DA optimum to achieve the maximum performance is different for each function, and hence for each DA-dependent task (Cai and Arnsten, 1997; Feil et al., 2010; Cools and D'Esposito, 2011). Consequently, during the pre-ovulatory phase, the peak in estradiol leads to a rise in DA levels and therefore modifies the performance depending on the individual differences in DA baseline levels. In this way, the effects of menstrual cycle on higher cognitive functions would not be consistent unless differences in DA baseline levels between women are accounted for. This hypothesis has been previously reported for the working memory during high control interference trials (*lure* trials) (Jacobs and D'Esposito, 2011). Women with low baseline DA levels (indicated by the enzyme catechol-O-methyltransferase, COMT) presented enhanced performance during the pre-ovulatory phase related to an increase in estradiol whereas women with higher DA levels presented impaired performance. However the role of progesterone has not been fully characterized. Given the modulating effect of progesterone on the dopaminergic system (Frye and Sora, 2010) and its influence on estrogen impact (Dluzen and Ramirez, 1987; Yu and Liao, 2000), it is necessary to extend this research to the luteal phase, in which both estradiol and progesterone levels are higher compared to menses. Furthermore, the modulation of sex hormone effects by the DA baseline levels has not been generalized to other DA-dependent executive functions so far.

In the present study we used a verbal N-back task as measure of working memory (Braver et al., 1997), and a Stroop task as measure of cognitive flexibility (Stroop, 1935). The variant used for the N-back was similar to the task employed by Jacobs and D'Esposito (2011). The N-back task allows to not only study the ability to manipulate and retain information (as working memory is often described), i.e., “updating” via the accuracy in detecting targets, but also the attentional control of interference via *lure* trials (Engle and Kane, 2003; Gray et al., 2003). For the Stroop task, we used a single trial version (Dalrymple-Alford and Budayr, 1966), which demands more flexible control mechanism than the classical block-wise presentation, since congruent, neutral and incongruent trials are randomly presented avoiding anticipation. Therefore, this task does not only allow for the assessment of “inhibitory control” via the interference effect, but also for “shifting” via overall performance on the task. Furthermore, we used two different conditions, Stroop word and Stroop color, in order to explore the semantic and the color naming interference and facilitation separately. The parallel distributed processing (PDP) model (Cohen et al., 1990; MacLeod and MacDonald, 2000) suggests that interference arises from the processing of the word and color in parallel, therefore allowing us to explore the more automatic process of reading words (Stroop word), in contrast to naming colors (Stroop color) (MacLeod and Dunbar, 1988).

In humans, direct measurement of individual DA levels is only possible through techniques such as positron emission tomography (PET) and single-photon emission computed tomography (SPECT) (Willeit et al., 2016). However, due to their invasiveness, other indirect markers are more commonly used. The spontaneous eye blink rate (EBR) has been suggested as a non-invasive indicator of striatal DA levels (Colzato and Hommel, 2014; see review Jongkees and Colzato, 2016). Its relation to dopamine levels is now well-established from animal, pharmacological and clinical studies. EBR correlates positively with dopamine levels in the caudate nucleus of non-human primates (Taylor et al., 1999). DA agonists and antagonists increase and decrease EBRs, respectively in animals (Korsgaard et al., 1985; Elsworth et al., 1991; Lawrence and Redmond, 1991; Kleven and Koek, 1996; Kaminer et al., 2011) and humans (Blin et al., 1990; Strakowski et al., 1996; Strakowski and Sax, 1998). Additionally, hypo-dopaminergic conditions like Parkinson's lead to a reduction of the spontaneous blink rate (Karson, 1983; Karson et al., 1984; Korosec et al., 2006), while hyper-dopaminergic conditions like schizophrenia lead to an increase of the spontaneous blink rate (Cheung et al., 1995; Chen et al., 1996). So far, several studies have used the EBR as a behavioral index of DA functioning, predicting performance in DA-dependent cognitive tasks (Dreisbach et al., 2005; Colzato et al., 2007, 2008, 2009; Chermahini and Hommel, 2010; Akbari Chermahini and Hommel, 2012; Dang et al., 2016). However, the usefulness of this indicator has not yet been established in menstrual cycle research. In the present study, spontaneous EBR was used as an indicator of baseline striatal DA levels as suggested by Jongkees and Colzato (2016) and Colzato and Hommel (2014).

In summary, the main goal of the current study is to extend the previous findings of DA dependent changes in cognitive performance across the menstrual cycle to the luteal cycle phase on the one hand and to a variety of executive functions on the other hand. Furthermore, we intend to demonstrate the usefulness of the EBR as DA indicator in menstrual cycle research. As demonstrated previously, we expect cognitive benefits in the pre-ovulatory phase compared to the menses phase, if EBR is low during menses; and impairment in those individuals with high EBR during menses for working memory functions. We hypothesize a similar interaction for inhibitory control and shifting of information as assessed with the Stroop task. Different DA optima will be explored by different EBR cut-offs. Regarding the luteal phase, increases or decreases in performance compared to the pre-ovulatory phase are expected and should shed light on the modulatory role of progesterone on the estrogenic actions in the dopaminergic system.

## Materials and methods

### Participants

Forty-three healthy right-handed women were recruited for the study on the campus of the Faculty of Sciences of the University of Salzburg and through social media. All participants were German-speaking and most of them university students. A total of 7 participants were excluded prior to analyses because of inconsistencies between hormone values and cycle phase as calculated based on self-reports. Therefore, analyses were performed in 36 women with an age range between 18 and 33 years (*M*_age_ = 23.36, *SD* = 3.44). All of them had a regular menstrual cycle (*M*_cycle length_ = 28.67 days, *SD* = 2.48) and had not used hormonal contraceptives within the previous 6 months. Regular menstrual cycle was defined as ranging between 21 and 35 days and a variability of cycle length between individual cycles of less than 7 days (Fehring et al., 2006). Other exclusion criteria were neurological, psychiatric or endocrine disorders, and being under medication treatment. All participants received either course credits or 30 € for their participation.

### Ethics statement

Experiments were conducted in accordance with the Code of Ethics of the World Medical Association (Declaration of Helsinki), and all participants gave their informed written consent to participate in the study. The institutional guidelines of the University of Salzburg (Statutes of the University of Salzburg - see https://online.uni-salzburg.at/plus_online/wbMitteilungsblaetter.display?pNr=98160) state in §163 (1) that ethical approval is necessary for research on human subjects if it affects the physical or psychological integrity, the right for privacy or other important rights or interests of the subjects or their dependents. In §163 (2) it is stated that it is the responsibility of the PI to decide, whether (1) applies to a study or not. Therefore, we did not seek ethical approval for this study. Since it was non-invasive and performed on healthy adult volunteers, who gave their informed consent to participate, (1) did not apply. Data were processed in anonymized/deidentified form. Upon arrival at the lab, participants were assigned a subject ID (VP001, VP002, etc.), which was used throughout the study.

### Procedure

In order to study every possible combination of hormonal levels, participants were tested in three sessions, time-locked to the subject's menstrual cycle as follows. First, during menses (low progesterone and estradiol); second in the pre-ovulatory phase (when estradiol levels peak and progesterone is still low), and last during the mid-luteal phase (high progesterone and estradiol), order counter-balanced. Menses phase spanned from the second day of menstruation to 7 days before ovulation (*M*_day_ = 3.92, *SD* = 2.34) depending on the individual cycle length. Pre-ovulatory appointments were scheduled 3 days before the expected ovulation (*M*_day_ = 11.93, *SD* = 3.07), which can be calculated subtracting 14 days to the length of the whole cycle, since luteal phase, the following period, is relatively stable among women (Fehring et al., 2006). The ovulation was confirmed by commercially available ovulation tests (Pregnafix® Ovulationstest), which test for the LH surge in urine. Mid-luteal phase ranged from day 3 post ovulation to 3 days before the onset of the next menstruation (*M*_day_ = 21.50, *SD* = 3.26), which was confirmed by follow up reports of the participants.

During each session participants filled in a brief questionnaire to assess food, sports and sleeping habits during the previous hours, and possible stressors within the last weeks. Afterwards EBR was measured and saliva samples taken as explained in the following sections. Then, both tasks were applied with a break in between them. EBR is supposed to be stable during daytime but increases in the evening (Barbato, 2000), and cortisol levels, which are known to interact with estradiol levels (Kirschbaum et al., 1999; Whirledge and Cidlowski, 2010), typically are highest in the morning, right after waking (Kirschbaum and Hellhammer, 1989; Pruesser et al., 1997; Schmidt-Reinwald et al., 1999). To avoid any confounds by these diurnal changes, data were always collected after 11 a.m. and before 5 p.m. and the three sessions of the same participants were scheduled approximately around the same hour.

### Cognitive paradigms

Stimulus presentation and data acquisition were done with Presentation® (Systems, 2011, Neurobehavioral Systems, http://www.neurobs.com/) in a CRT monitor (1,024 × 768 pixel resolution, 120 Hz refresh rate). As part of a larger study, participants completed two different tasks:

#### N-back task

**Figure 1:**
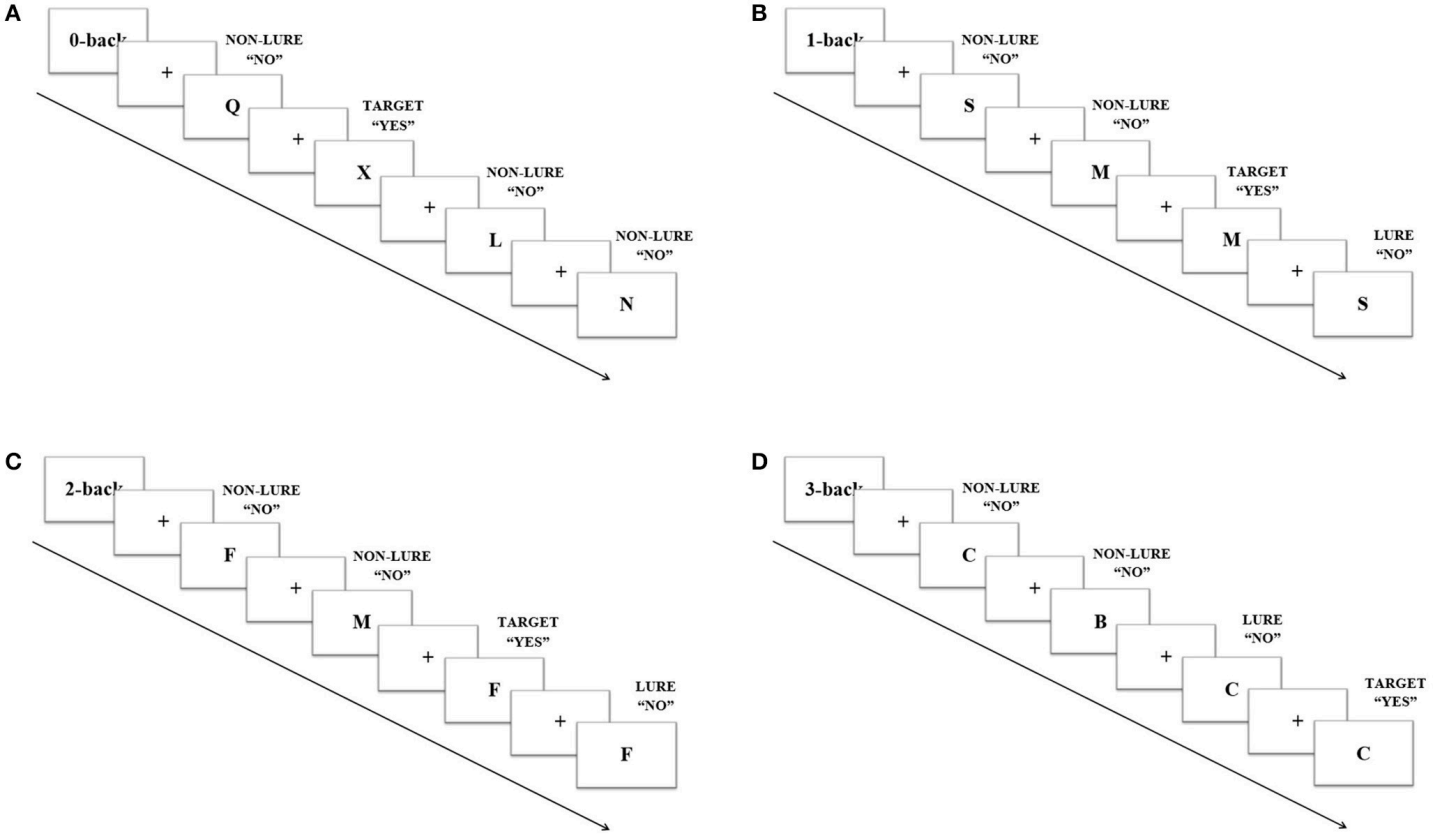
Example of four trials in the N-back task. Every trial appeared every 2 s and lasted 1 s each. Participants were instructed to press left mouse button when “yes” and the right one if “no.” For 0-back **(A)**, targets were letter X; for 1-back **(B)**, the target was when the letter matched the previous letter; and for 2-back **(C)** and 3-back **(D)** when it was identical to the one presented 2 or 3 trials before, respectively.

The N-back verbal task consisted of 4 levels of load: 0-, 1-, 2-, and 3-back; and 3 different trial types: targets, lures and non-lures. Upper case black letters were presented on a white background. Participants were instructed to respond to each letter by pressing the left mouse button when the answer was “yes” and the right one if it was “no.” In the 0-back level participants indicated whether or not the target letter X appeared. In this condition there were not lures, trial types consisted only of targets and non- targets. For the 1-back level the participants responded whether the letter matched the previous letter, and for 2- and 3-back whether it was identical to the one presented 2 or 3 trials before, respectively (Figure 1). Three different versions of N-back task were presented, one in each session, order counterbalanced. Each version consisted of 16 blocks of trials (4 blocks of each level) ordered in a Latin squares sequence. Every block included 20 stimuli which appeared every 2 sec and lasted 1 s each. Trials were pseudo-randomly ordered and their proportions were 20% targets, 65% non-lures, and 15% lures, except for the 0-back, in which targets were 20% and non-lures 80%. Blocks were preceded by 6 s of instructions each and separated from one another by 16 s (including instructions). The whole task was always preceded by a training version which included one shortened block of each level.

Accuracy and response time were recorded for each trial type and condition. Additionally, for the targets, as a measure of sensitivity, d prime (d') was calculated for all 4 n-back conditions, based on the signal detection theory (Swets et al., 1961). This discriminability index has been proposed to provide a more suitable index of working memory performance, and to be less susceptible to demographic variables (Haatveit et al., 2010). It describes the relationship between the signal and noise distributions and therefore offers information about the participant's discrimination between targets and non-targets (Wickens, 2001, p. 3–16). For its calculation we followed the formula d' = Z_Hit_ – Z_FA_ (Macmillan and Creelman, 1990). The hit rate (Hit) is expressed by the proportion of correct answers to targets when they appear (hits target/number of targets), and the false alarm rate (FA), is expressed by the incorrect answers to non-targets, which include both lures and non-lures (incorrect non-target/number of non-targets). To adjust the perfect scores, values were replaced as previously reported by Haatveit et al. (2010), by 1−1/(2n) for perfect hits and 1/(2n) for zero false alarms, with n being the total number of targets (16) or non-targets (64). Following these formulas higher values of d' (ranging between ±4.28) indicate higher sensitivity.

#### Stroop task

**Figure 2:**
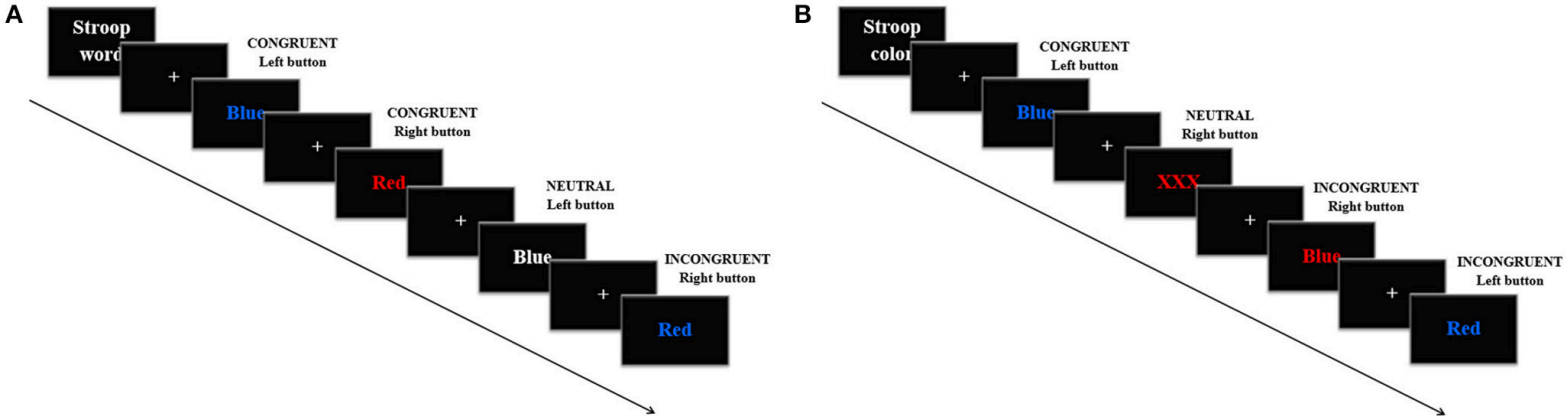
Example of four trials in the Strop task. Every trial appeared every 1.5 s. In the Stroop word **(A)**, participants were told to press the left mouse button when the word “Blue” was presented and the right one when the word “Red” was presented. In the Stroop color **(B)**, participants were asked to press the left mouse button when the letters were written in blue and the right one when the letters were written in red.

The Stroop task consisted of an adapted single trial version divided in a color and a word variant. Each condition of the task included three blocks in which 120 trials were presented in a random order every 1.5 s. In the Stroop word, the words “Blue” and “Red” appeared in red, blue or white color. In this condition, participants were told to press the left mouse button when the word “Blue” was presented and the right one when “Red” was, in this case regardless the color of the letters. There were also three trial types: *congruent*, when “Blue” was written in blue color or when “Red” was written in red color; *incongruent*, when “Blue” was written in red or when “Red” was written in blue; and *neutral*, when any of the words appeared written in white (Figure 2A). The proportions of the trials were: 33% congruent, 33% incongruent, and 33% neutral, all of the groups with half of the trials with the word “Blue” and half with the word “Red.” In the Stroop color, the words “Blue,” “Red,” or the string “XXX” appeared in uppercase letters on a black background either on blue or red color. Participants were asked to press the left mouse button when a string of letters written in blue appeared and the right one when it was red, always regardless the meaning of the word. There were three trial types: *congruent*, when “Blue” was written in blue color or when “Red” was written in red color; *incongruent*, when the former words were written in a mismatching color; and *neutral*, when the string “XXX” appeared (Figure 2B). The proportions of the trials were: 33% congruent, 33% incongruent, and 33% neutral, half red half blue in every group. For both conditions, participants completed a training version, which consisted of a shortened block with 12 trials in the same proportions as described.

### EBR measurement

Spontaneous EBR was taken as an indirect measure of baseline DA levels. Fluctuations in the DA levels and hence in the EBR can be found related to estradiol levels. A decrease in EBR in older women has been related to an estradiol decline (Chen et al., 2003), and oral contraceptives are suggested to increase the EBR (Yolton et al., 1994). Therefore, we used the EBR recorded during menses as DA indicator, in order to ensure it was not affected by any hormonal influences.

For the recording, subjects were seated 1 m from a white wall with a black cross at their eyes height. They were left alone in the room and asked to fix their gaze at the cross silently and in resting conditions. Vertical and horizontal electro-oculograms (EOGs) were recorded with an EEG system (actiCAP, Brain Products GmbH, Germany) at a sampling rate of 500 Hz and impedances kept under 50 kΩ. Skin electrodes were placed above and below the right orbita, and at the outer canthi, referenced against the vertex electrode (Cz), and a grounding electrode was located on the forehead. An eye blink was defined as a sharp high amplitude wave with a voltage change of 100 uv in a time interval of 500 ms. The EBR was defined as the number of blinks per minute as averaged over six consecutive minutes. Signals were amplified using an ActiCHamp Amplifier (Brain Products GmbH, Germany). The analysis of the recorded blinks was performed online with Brain Vision Analyzer 2.1(Brain Products™ GmbH, Munich, Germany), in which two independent observers visually scored the number of blinks for the 6 min segment. The inter-rater agreement was 100%. The EBR during menses ranged from 2.00 to 42.33 blinks per min (*M*_EBR_ = 16.14, *SD* = 10.73). This large spectrum, although indirectly, should provide the sufficient variability in dopaminergic activity between subjects.

### Hormone analysis

In order to assess estradiol and progesterone levels three saliva samples 2 ml each were collected throughout every session: one before the tasks, one after, and one in between. Until further analysis they were stored in a freezer at −20°C and solid particles were removed by centrifugation (3,000 rpm for 15 min, then 3,000 rpm for 10 min). For hormone analyses, the three samples were pooled in order to control for diurnal fluctuations in hormone levels and to ensure reliability of hormone assessment. Estradiol and progesterone levels were quantified using ELISA kits from DeMediTec Diagnostics which are based on the competition principle and microplate separation using polyclonal antisera coated to the wells. Sensitivity is 0.6 pg/ml for estradiol and 5.0 pg/ml for progesterone. Intra-assay variation (CV) is between 2.4 and 8.3% for estradiol and between 6.0 and 9.6% for progesterone. Inter-assay variation (CV) is between 2.8 and 12.0% for estradiol and between 8.6 and 10.1% for progesterone.

### Statistical analysis

Those sessions in which overall performance was below chance were excluded from the analysis. In particular this concerned two whole sessions from 2 different participants in the Stroop task, and every session from one participant in the N-back task. Statistical analyses were carried out in R 3.2.2., assessing the possible effect of the factors on each dependent variable through linear mixed models as follows. First, we used the *lmer* function of the *lme4* package, modulating the participant number as random factor, so that we controlled for repeated measurements. In every analysis, the factors that were not relevant to explain the model were eliminated with the *step* function of the *lmerTest* package at its default settings. The specific models are described in the respective paragraph of the results section. In all models, both the dependent and continuous independent variables were z-standardized using the scale function. Therefore, the coefficients b of fixed effects in the models represent a standardized effect size based on standard deviations, similar to Cohen's d.

## Results

### Cycle phase and hormone levels

In order to compare sex hormone levels between the different cycle phases, the fixed factor cycle phase was modeled for dependent variables estradiol and progesterone, respectively in the context of a linear mixed effects model.

Estradiol was significantly higher in the pre-ovulatory phase compared to menses phase [*b* = 0.17, *SE*_b_ = 0.06, *t*_(35)_ = 3.06, *p* < 0.01] and in luteal phase compared to menses phase [*b* = 0.27, *SE*_b_ = 0.10, *t*_(35)_ = 2.83, *p* < 0.01] and did not differ significantly between pre-ovulatory and luteal phases. Progesterone was significantly higher in the luteal phase compared to the pre-ovulatory phase [*b* = 0.33, *SE*_b_ = 0.09, *t*_(33)_ = 3.54, *p* < 0.05] and in luteal phase compared to the menses phase [*b* = 0.41, *SE*_b_ = 0.11, *t*_(34)_ = 3.89, *p* < 0.001] and did not differ significantly between pre-ovulatory and menses (all means are displayed in Table 1).

**Table 1:** Mean levels of salivary estradiol and progesterone in each cycle phase.

	**Estradiol (pg/ml)**		**Progesterone (pg/ml)**	
	***M***	***SD***	***M***	***SD***
Menses	2.13	0.59	88.74	96.72
Pre-ovulatory	2.34	0.62	124.79	159.32
Luteal	2.61	1.06	290.76	303.80

*M, Mean; SD, Standard deviation*.

### N-back task

Since targets and lures assess functionally different processes, i.e., updating and inhibition, they were analyzed separately (Engle and Kane, 2003; Gray et al., 2003). For targets, linear mixed models were applied to RT, accuracy and sensitivity as dependent variables. For lures, linear mixed models were applied to RT and accuracy as dependent variables. For both, targets and lures, we included the participant number (PNr) as random factor and session on the one hand, as well as the interactive effects of load, cycle phase and EBR during menses on the other hand, as fixed variables (e.g., RT ~ 1|PNr + session + load^*^cycle^*^EBR). Load, session and EBR were included as continuous variables, while cycle phase was factorized, which allows the assessment of differences between the pre-ovulatory phase and menses on the one hand and the luteal phase and menses on the other hand. In order to assess differences between luteal phase and pre-ovulatory phase, all models were rerun after excluding menses. Thus, for each dependent variable, in the first model the high hormone phases were compared to menses, while in the second model, the luteal phase was compared to the pre-ovulatory phase.

#### Interactive effects of cycle phase and EBR during menses

##### Targets

*Accuracy*. For the accuracy with targets, the main effect of session was non-significant and thus removed from the models. We observed a significant main effect of load [*b* = −0.46, *SE*_b_ = 0.04, *t*_(380)_ = −11.88, *p* < 0.001], indicating that accuracy was reduced as the load increased. Furthermore, the main effects of cycle phase and EBR were not significant and did not interact with each other or with load in both models. Therefore, they were removed from the models.

**Figure 3:**
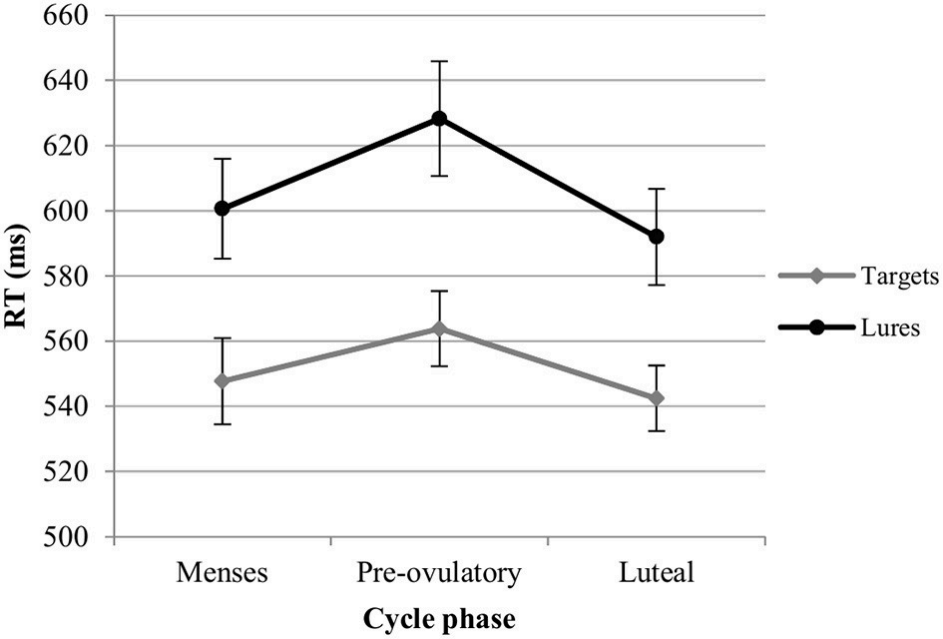
Reaction time (RT) for the N-back task along the menstrual cycle. Responses were significantly faster in the luteal phase compared to pre-ovulatory phase for both targets and lures.

*RT*. For the RT with targets, we observed significant main effects of session [*b* = −0.19, *SE*_b_ = 0.03, *t*_(379)_ = −5.48, *p* < 0.001] and load [*b* = 0.52, *SE*_b_ = 0.03, *t*_(379)_ = 15.20, *p* < 0.001]. RT became shorter with the number of sessions, and longer as the load increased. In the first model, luteal and pre-ovulatory phases did not differ from menses and cycle phase is removed from the model. In the second model, women were significantly faster in their answers to targets during the luteal phase compared to the pre-ovulatory phase [*b* = −0.16, *SE*_b_ = 0.08, *t*_(241)_ = −2.15, *p* < 0.05; Figure 3]. The main effect of EBR was not significant in both models and did not interact with the cycle effects or load. Therefore, it was removed from the models. The cycle effect did also not interact with load.

*Sensitivity*. For the sensitivity with targets, we observed significant main effects of session [*b* = 0.11, *SE*_b_ = 0.03, *t*_(362)_ = 3.19, *p* < 0.01] and load [*b* = −0.65, *SE*_b_ = 0.03, *t*_(362)_ = −18.96, *p* < 0.001], indicating that sensitivity was increased along the sessions and reduced as the load increased. The main effects of cycle phase and EBR were not significant and did not interact with each other or with load in both models and were, therefore, removed from the models.

##### Lures

*Accuracy*. For the accuracy with lures, we observed significant main effects of session and load. Performance increased with the number of sessions [*b* = 0.09, *SE*_b_ = 0.04, *t*_(261)_ = 2.04, *p* < 0.05], and decreased with higher levels of load [*b* = −0.57, *SE*_b_ = 0.04, *t*_(261)_ = −13.73, *p* < 0.001]. The main effects of cycle phase and EBR were not significant and did not interact with each other or with load in both models. Therefore, they were removed from the models.

*RT*. For the RT with lures, we observed significant main effects of session [*b* = −0.25, *SE*_b_ = 0.04, *t*_(259)_ = −5.99, *p* < 0.001] and load [*b* = 0.40, *SE*_b_ = 0.41, *t*_(259)_ = 9.92, *p* < 0.001]. RT became shorter along the sessions and longer as the load increased. In the first model, luteal and pre-ovulatory phases did not differ from menses and cycle phase is removed from the model. In the second model, we found a significant difference between luteal and pre-ovulatory phase [*b* = −0.23, *SE*_b_ = 0.10, *t*_(161)_ = −2.24, *p* < 0.05]. Women were faster in responding to the lures during the luteal phase as compared to the pre-ovulatory phase (Figure 3). The main effect of EBR was non-significant and did not interact with load or the cycle effects in both models. Therefore, it was removed from the models. The cycle effect did also not interact with load.

#### Effect of estradiol and progesterone levels

To explore, whether the cycle effects observed for the RT were attributable to estradiol or progesterone, the final models from above were rerun, replacing cycle phase by estradiol and progesterone values, respectively (RT ~ 1|PNr + session + load + hormone).

##### Targets

**Figure 4:**
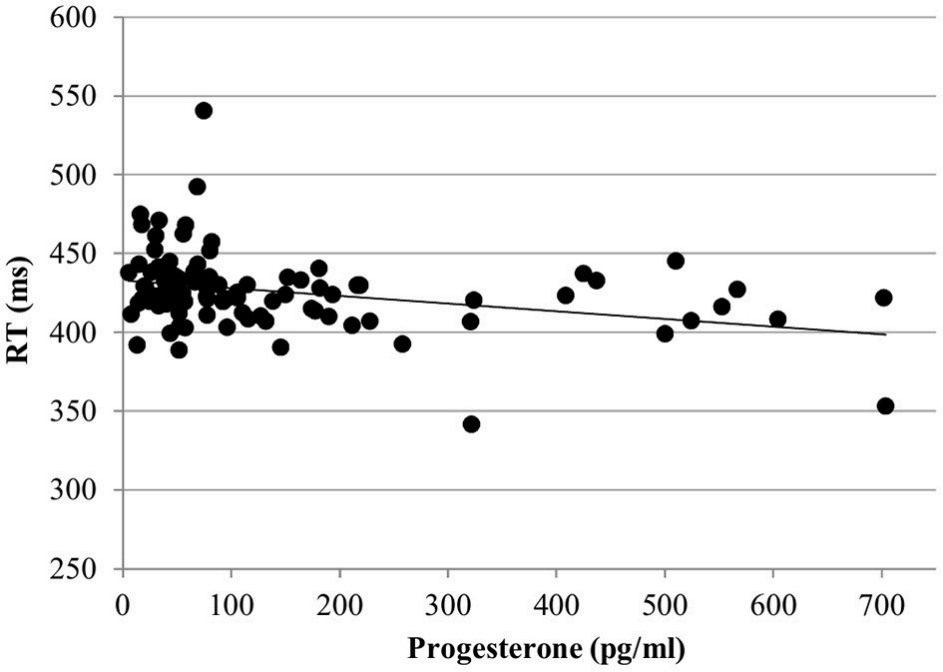
Relationship of progesterone levels to reaction time (RT) for the targets of the N-back task. Responses to targets were slower the lower the progesterone levels of women.

For the RT with targets, no effect of estradiol was found. However, we found a significant main effect of progesterone [*b* = −0.09, *SE*_b_ = 0.04, *t*_(363)_ = −2.17, *p* < 0.05]. The lower the progesterone levels of women, the slower were their reactions to targets (Figure 4).

##### Lures

Both estradiol and progesterone did not affect the RT with lures, so they were removed from the model.

### Stroop task

For the Stroop task, word and color condition were analyzed separately, as they assess qualitatively different processes (MacLeod and Dunbar, 1988; Cohen et al., 1990). For both conditions, linear mixed models were applied to RT and accuracy as dependent variables, including the participant number (PNr) as random factor and session as well as the interactive effects of trial type, cycle phase and EBR as fixed factors (e.g., RT ~ 1|PNr + session + trial^*^cycle^*^EBR). Session and EBR were included as continuous variables, while trial type and cycle phase were factorized. Regarding trial type this allows for the assessment of interference effects (incongruent vs. neutral) on the one hand and facilitation effects (congruent vs. neutral) on the other hand. Regarding cycle phase, this allows the assessment of differences between the pre-ovulatory phase and menses on the one hand and the luteal phase and menses on the other hand. All models were rerun after excluding menses, allowing us to assess the differences between luteal phase and pre-ovulatory phase. Therefore, for each dependent variable, high hormone phases were compared to menses in the first model, whereas the luteal phase was compared to the pre-ovulatory phase in the second model.

#### Interactive effect of cycle phase and EBR during menses

##### Word condition

*Accuracy*. For the accuracy in the word condition, the main effect of session was non-significant and thus removed from the models. We found a significant main effect of trial type. The accuracy was significantly lower for the incongruent trials compared to the neutral ones [Interference effect; *b* = −0.32, *SE*_b_ = 0.10, *t*_(280)_ = −3.07, *p* < 0.01], but did not differ significantly between congruent and neutral trials [Facilitation effect; *b* = 0.08, *SE*_b_ = 0.10, *t*_(280)_ = 0.77, *p* = 0.44]. The main effects of cycle phase and EBR were non-significant and did not interact with trial type or each other and were thus removed from the model.

**Figure 5:**
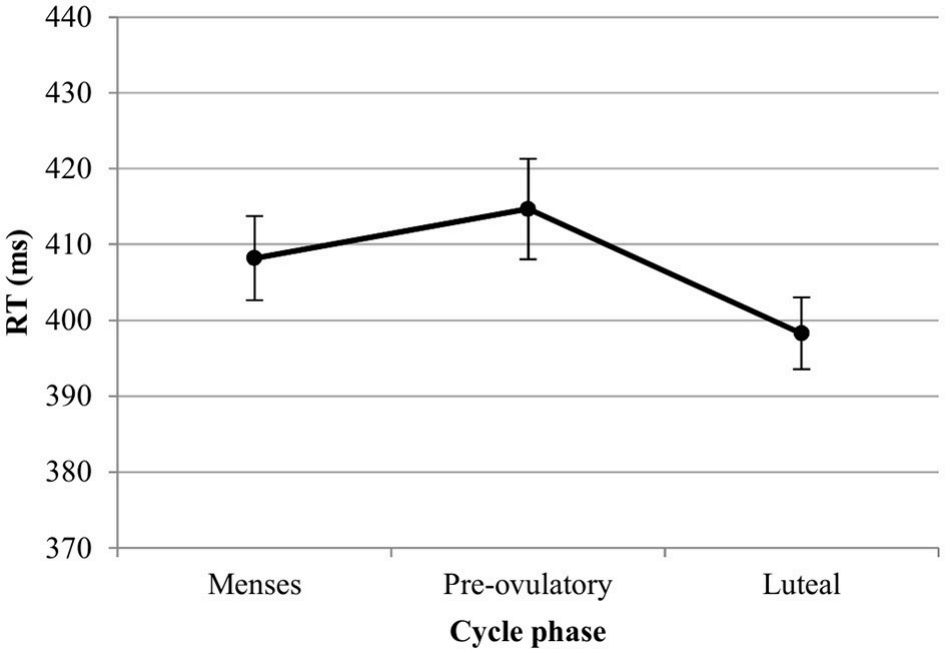
Reaction time (RT) for the Stroop word along the menstrual cycle. Responses were significantly faster in the luteal phase compare to menses and pre-ovulatory phase.

*RT*. For the RT in the word condition, we observed significant main effects of session [*b* = −0.15, *SE*_b_ = 0.03, *t*_(277)_ = −5.14, *p* < 0.001] and trial type. RT increased significantly along the number of sessions and were significantly slower for incongruent trials compared to neutral trials [Interference effect; *b* = 0.17, *SE*_b_ = 0.07, *t*_(277)_ = 2.50, *p* < 0.05]. There was no significant difference between RT for congruent and neutral trials [Facilitation effect; *b* = −0.06, *SE*_b_ = 0.07, *t*_(277)_ = −0.84, *p* = 0.40]. Furthermore, there was a significant main effect of phase on the RT, which did not interact with trial type. In the first model, we found a significant difference between luteal phase and menses [*b* = −0.18, *SE*_b_ = 0.07, *t*_(277)_ = −2.63, *p* < 0.01], while the pre-ovulatory phase and menses did not differ significantly [*b* = 0.12, *SE*_b_ = 0.07, *t*_(277)_ = 1.79, *p* = 0.07]. In the second model, we found a significant difference between luteal and pre-ovulatory phase [*b* = −0.31, *SE*_b_ = 0.07, *t*_(173)_ = −4.42, *p* < 0.001]. Women were significantly faster in responding during the luteal phase as compared to menses and the pre-ovulatory phase, irrespective of the type of trial (Figure 5). The main effect of EBR was non-significant and did not interact with trial type or phase and was thus removed from the model.

##### Color condition

**Figure 6:**
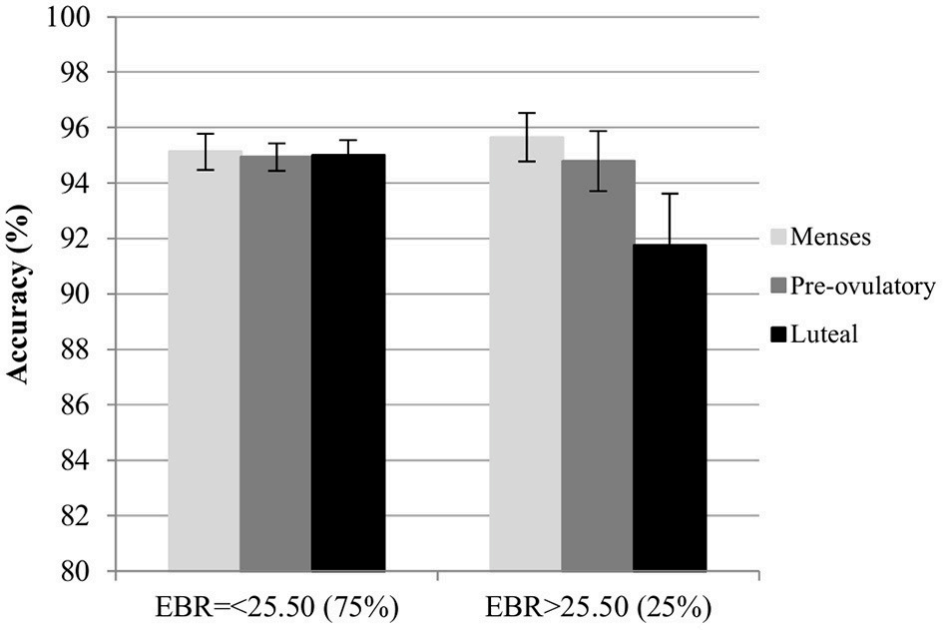
Mean accuracy for the Stroop color along the menstrual cycle. Performance was worse in global in the luteal phase compare to menses and pre-ovulatory for women with higher EBR during menses.

*Accuracy*. For the accuracy in the color condition, significant main effects of session and trial type were found. Accuracy increased significantly with the number of sessions [*b* = 0.13, *SE*_b_ = 0.05, *t*_(275)_ = 2.73, *p* < 0.01]. Among the trial types, accuracy was significantly lower for incongruent trials compared to neutral trials [Interference effect: *b* = −0.25, *SE*_b_ = 0.11, *t*_(275)_ = −2.29, *p* < 0.05], whereas accuracy for congruent trials was significantly higher than for neutral trials [Facilitation effect: *b* = 0.22, *SE*_b_ = 0.11, *t*_(275)_ = 2.02, *p* < 0.05]. The main effects of cycle phase were non-significant in both models (all |*b*| < 0.22, all *SE*_b_ < 0.12, all *t* < 1.92, all *p* > 0.05), as was the main effect of EBR [*b* = −0.03, *SE*_b_ = 0.12, *t*_(34)_ = −0.27, *p* = 0.79]. Also, the interactions between trial type and cycle phase as well as trial type and EBR were non-significant and thus removed from the model. There was a significant modulation of the accuracy by the interaction of the EBR during menses and the cycle phase of participants in both models. This interaction was driven by changes during the luteal phase compared to menses [*b* = −0.27, *SE*_b_ = 0.11, *t*_(275)_ = −2.47, *p* < 0.05] in the first model, while it was non-significant for the comparison of pre-ovulatory phase and menses [*b* < 0.004, *SE*_b_ = 0.11, *t*_(275)_ = −0.003, *p* > 0.99]. The interaction was also confirmed for the comparison of luteal phase to the pre-ovulatory phase in the second model [*b* = −0.25, *SE*_b_ = 0.11, *t*_(172)_ = −2.20, *p* < 0.05]. Irrespective of the number of sessions or the type of trial, in the luteal phase accuracy decreased more strongly the higher the EBR during menses (Figure 6).

**Figure 7:**
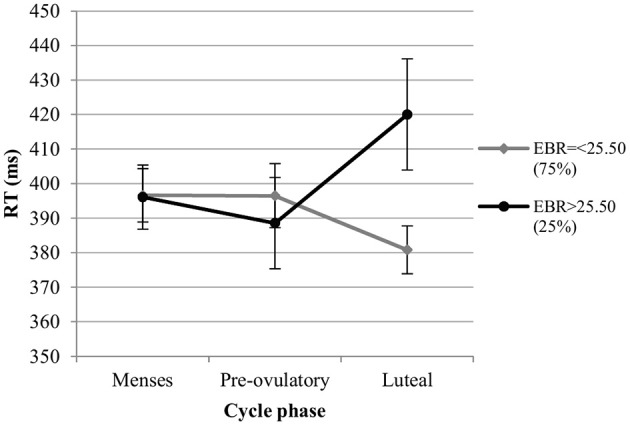
Interactive effects of cycle phase and EBR on reaction time (RT) for Stroop color. Responses were slower in the luteal phase compare to menses and pre-ovulatory phase for women with higher EBR during menses and faster in the luteal phase compare to the menses and pre-ovulatory phase for women with lower EBR during menses.

*RT*. For the RT in the color condition we also observed significant main effects of session and trial type. RT decreased significantly with the number of sessions [*b* = −0.13, *SE*_b_ = 0.03, *t*_(275)_ = −4.34, *p* < 0.001], and were significantly longer for incongruent trials compared to neutral trials [Interference effect; *b* = 0.27, *SE*_b_ = 0.07, *t*_(275)_ = 3.81, *p* < 0.001], while RT did not differ significantly between congruent and neutral trials [Facilitation effect; *b* < 0.004, *SE*_b_ = 0.07, *t*_(275)_ = −0.04, *p* = 0.97]. The main effect of cycle phase was non-significant in the first model [all |*b*| < 0.10, all *SE*_b_ < 0.08, all *t*_(275)_ < 1.40, all *p* > 0.05], as was the main effect of EBR [*b* = −0.06, *SE*_b_ = 0.15, *t*_(34)_ = −0.39, *p* = 0.70]. The interactions between trial type and cycle phase as well as trial type and EBR were also non-significant and thus removed from the model. There was a significant phase^*^EBR interaction in the first model, which was attributable to changes during the luteal phase compared to menses phase [*b* = 0.26, *SE*_b_ = 0.07, *t*_(275)_ = 3.68, *p* < 0.01], while no differences were observed between the pre-ovulatory phase and menses phase [*b* = 0.14, *SE*_b_ = 0.07, *t*_(275)_ = 1.94, *p* = 0.05]. During the luteal phase, the RT was shorter compared to the menses phase for women with lower EBR during menses, whereas it was significantly longer compared to the menses phase for those with higher EBR during menses (Figure 7). In the second model, the main effect of cycle phase was non-significant and did not interact with EBR or trial type, indicating that luteal and pre-ovulatory phases did not differ from each other. It was thus removed from the model.

As different DA optima have been observed for different tasks, additional analyses were carried out to approximate the EBR cut off responsible for the interactions observed. The sample was split into groups by the quartiles of EBR during menses. The cut-off at which the interaction effects between EBR during menses and cycle phase disappeared was determined, by successively removing quartiles bottom up.

*Accuracy*. For the accuracy, the interaction remained in the upper 75% after removing the first quartile [*b* = −0.29, *SE*_b_ = 0.13, *t*_(206)_ = −2.30, *p* < 0.05] and in the upper 50% after removing the first two quartiles [*b* = −0.44, *SE*_b_ = 0.15, *t*_(134)_ = −3.01, *p* < 0.01]. However, the interaction disappeared from the model when only considering the upper quartile. Therefore, the cut off in the EBR for the accuracy in the Stroop color lay between the 50% and the 75% quartiles.

*RT*. For the RT, the interaction remained significant even in the highest quartile [*b* = 0.54, *SE*_b_ = 0.13, *t*_(62)_ = 4.01, *p* < 0.001]. Consequently, for the RT of the Stroop color, the cut off lay within the upper 25%.

#### Effect of estradiol and progesterone levels

To determine whether the effects of cycle phase observed above were attributable to estradiol or progesterone, the final models from above were rerun, replacing cycle phase by estradiol and progesterone values respectively (word: RT ~ 1|PNr + session + trial type + hormone, color: e.g. RT ~ 1|PNr + session + trial type + hormone^*^EBR).

##### Word condition

**Figure 8:**
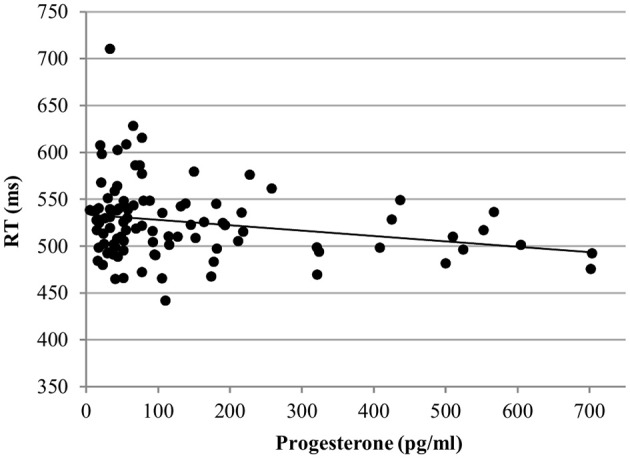
Relationship of progesterone levels to reaction time (RT) for Stroop word. Responses were faster the higher the progesterone levels of women.

*RT*. In the word condition, only progesterone, but not estradiol affected the RT [*b* = −0.12, *SE*_b_ = 0.04, *t*_(267)_ = −3.27, *p* < 0.01]. Irrespective of any other factor, participants were faster the higher their levels of progesterone (Figure 8).

##### Color condition

*Accuracy*. Both estradiol and progesterone did not affect accuracy and there were no interactions between EBR and estradiol or progesterone in the analysis of accuracy, so every factor was removed from the model.

**Figure 9:**
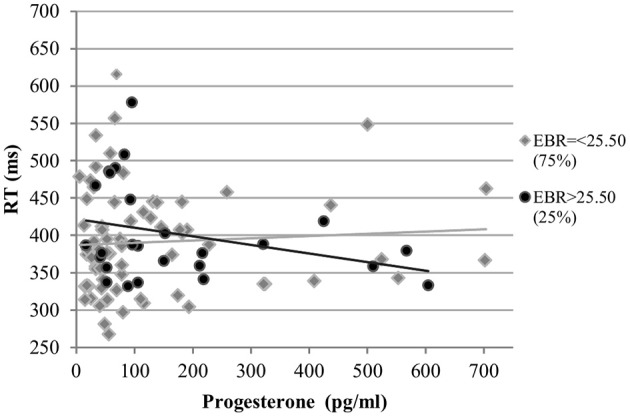
Interactive effects of progesterone and EBR on reaction time (RT) for Stroop color. Women with lower EBR during menses were slower the higher their progesterone levels, whereas women with higher EBR during menses were faster as their progesterone levels increased.

*RT*. The RT was also not affected by estradiol levels and estradiol did not interact with EBR in the analysis of RT. The main effects of progesterone levels and EBR were non-significant [*b* = 0.02, *SE*_b_ = 0.04, *t*_(266)_ = 0.54, *p* = 0.59; *b* = 0.07, *SE*_b_ = 0.15, *t*_(33)_ = 0.45, *p* = 0.65, respectively]. However, RT was modulated by a significant interaction of progesterone with EBR during menses [*b* = 0.13, *SE*_b_ = 0.05, *t*_(266)_ = 2.70, *p* < 0.01]. This way, women with lower EBR during menses were slower the higher their progesterone levels, whereas women with higher EBR during menses were faster as their progesterone levels increased (Figure 9).

## Discussion

The primary goal of this study was to investigate the modulating effect of DA baseline levels on menstrual cycle changes in DA dependant processes. On the one hand, we wanted to extend previous findings to (i) the luteal cycle phase and (ii) a variety of cognitive functions using different tasks. On the other hand, we wanted to explore the usefulness of the EBR as a non-invasive indicator of DA baseline levels in menstrual cycle research. To address these aims, we tested women during three cycle phases—menses (low sex hormones), pre-ovulatory (high estradiol and low progesterone), and luteal phase (high estradiol and progesterone)—on two different tasks, the N-back and the Stroop task, while assessing their EBR during menses.

In the N-back task for both targets (updating) and lures (inhibition), women were significantly faster during the luteal phase compared to the pre-ovulatory phase. For the targets (updating) this effect was related to the progesterone levels, i.e., women were faster, the higher their progesterone level. Similarly, for the Stroop word condition, women were significantly faster during the luteal phase as compared to menses and the pre-ovulatory phase. This effect was also related to progesterone levels, as participants were faster the higher their levels of progesterone. However, neither in the N-back task nor in the Stroop word task did we find an effect of cycle phase on accuracy. Thus, during the luteal phase women increased their response speed with no change in accuracy, which is indicative of better performance in both tasks. We interpret these findings as an evidence of improved verbal skills during the luteal phase compared to other phases. Both the verbal N-back task and Stroop word condition involve inner speech, and it is well documented that silent repetition of letters and single word reading tasks engage similar brain regions such as left inferior frontal gyrus (including BA 44, Broca's area) and other peri-sylvian regions (Paulesu et al., 1993; see Turkeltaub et al., 2002 for a meta-analysis). Our findings are consistent with previous reports, demonstrating enhanced verbal abilities during the luteal phase, including simple verbal output tasks (Hampson, 1990; Maki et al., 2002; Šimič and Santini, 2012). Our results indicate that this effect is attributable to progesterone, but not estradiol as suggested by previous studies (Maki et al., 2002). A study from Natale et al. (2001) found that in postmenopausal women, the addition of progestogens to estrogen therapy replacement, improved verbal memory compared to estrogen alone.

Contrary to our expectations we have not been able to replicate the DA-cycle phase interaction reported by previous studies for the lure trials of the N-back task (Jacobs and D'Esposito, 2011). One of the main reasons for this may be the methodological differences to distinguish high and low DA baseline levels (genotyping vs. EBR). In the study of Jacobs and D'Esposito (2011) the DA baseline level was indexed by the COMT genotype and its activity levels. This enzyme metabolizes DA, and its efficiency depends on a polymorphism (Val^158^Met) for the gene that encodes it. Specifically, the *val/val* allelic variant is more efficient compare to the *met/met* form and therefore leading to lower DA baseline levels. In this study, we used the EBR during menses as a continuous variable instead of splitting the sample in fixed groups. However, this variable doesn't allow us to directly infer the DA levels and we can just compare the individuals within the range sampled, without knowing where exactly they are situated in the normal Gaussian curve.

We did however find an interaction between cycle phase and EBR during menses for the Stroop color condition. As the effect concerns the overall RT and accuracy, rather than the interference effect, it can be interpreted as an effect on the “shifting” function, or cognitive flexibility, rather than the “inhibitory control” function. In contrast, this interaction was not present in the Stroop word condition. These different findings can be understood in the context of the parallel distributed processing (PDP) model as explained in the introduction (Cohen et al., 1990; MacLeod and MacDonald, 2000). Reading the name of the colors (word condition) is more practiced and automatic than naming the color of words (color condition). Therefore, it is harder to inhibit the word reading in the color condition than inhibiting the color naming in the word condition. Consequently, the color condition elicits more response competition and requires more cognitive control than the word condition. This may explain why the DA-cycle interaction was found for the color, but not for the word condition.

Contrary to what we expected, the interaction effect in the color condition was driven by the luteal and not the pre-ovulatory phase. During the luteal phase, accuracy was lower and responses slower for women with high EBR during menses. This could have been explained by the higher values of estradiol during this phase, which, as it increases DA, would drive the DA level to exceed the optimal level for this task in women with already high DA baseline levels. However, we did not find our results to be related to estradiol levels, but to progesterone levels for RT. Women with higher EBR during menses were faster the higher their progesterone levels. This is counter-intuitive given that the same women show decreased performance during the luteal cycle phase, when progesterone levels peak. Thus, the increase in response times during the luteal cycle phase cannot be explained by the increased progesterone levels during that phase.

If progesterone exerted a positive effect on DA baseline levels like estradiol, we would expect an interaction in the opposite direction. As this is not the case, the most plausible explanation for our findings is that progesterone exerted a negative effect on DA baseline levels, thereby counteracting the positive effect of estradiol. Multiple findings support a differential effect of estradiol and progesterone on the central nervous system, although their combinatory effect is not clear. Specifically in the dopaminergic system, there is evidence for different modes of action between the two steroids (Morissette et al., 1990), combinatory but not synergistic effects (Morissette and Paolo, 1993), and opposite effects (Fernández-Ruiz et al., 1990). If progesterone counteracted the influence of estradiol on DA levels, this could have masked the relationship between estradiol and performance, and could explain why our findings don't relate to estradiol levels in a significant manner. Note however, that this could also be the result of a power failure or other factors driving the menstrual cycle effect that weren't taken into account in the present study. Furthermore, also note that the effect size of this interaction with progesterone was rather small and should therefore be interpreted with caution.

Currently, the literature on the effects of progesterone on the dopaminergic system in humans and its interaction with estradiol is inconclusive (Sun et al., 2016). This is also reflected by the discrepancies found in studies on executive functions. As an example, an improved performance for prefrontal executive control functions during the early luteal phase has previously been reported using the Wisconsin Card Sorting Test (Solis-Ortiz et al., 2004). In this EEG study they related their findings with the higher levels of progesterone and the role of this hormone in tasks demanding inner attention and cognitive control. In contrast, in the study by Hatta and Nagaya (2009) using the Stroop task, women showed and impaired performance during luteal phase compared to menses. These inconsistences may result from a lack of control for DA baseline levels.

The most important limitation of the present study, that may also explain why we were not able to find an effect of estradiol on performance, is that we did not adequately capture the pre-ovulatory peak of estradiol. Although estradiol was higher in the pre-ovulatory phase compared to menses, we found the highest estradiol values during the luteal phase, when progesterone is also high. Potential reasons for this are that estradiol levels fluctuate along the day due to the circadian rhythm (Shirtcliff et al., 2000), between different cycles (Becker et al., 2005) and sometimes exhibits different pattern from woman to woman (Gandara et al., 2007). In addition, insufficient analytical accuracy in individual assays have already been reported (Gao et al., 2015) and other studies have also failed to replicate the expected pattern of estradiol along the menstrual cycle in salivary samples, although the correlation with serum estradiol was high (Lu et al., 1999). One explanation for this could be inter-individual differences in the metabolism of estradiol within the salivary glands (Lu et al., 1999). However, since previous studies demonstrating interactive effects of estradiol and DA baseline levels on higher cognitive functions did not include the luteal cycle phase (Jacobs and D'Esposito, 2011), it is unclear whether the pre-ovulatory estradiol levels reported in their study were significantly higher than in the luteal phase.

In summary, we could not replicate previous results of DA-cycle interactions in the N-back task, but we found this interaction in the Stroop color condition. Thereby we were able to extend the idea of DA-cycle interactions to other cognitive functions, such as inhibitory control and shifting processes involved in this condition. Unlike previous studies, the inclusion of the luteal cycle phase also allowed us to relate the interactive effects of menstrual cycle and DA baseline levels to progesterone levels. The evidence presented here suggests that, not only estradiol, but the luteal phase and progesterone should also be considered in research on DA-cycle interactions. Last but not least, we have successfully identified the EBR as a useful indicator to differentiate baseline DA function in menstrual cycle research.

## Author contributions

BP designed and made the concept of the study. EH-L was responsible for data acquisition. Analysis and interpretation of the data were performed by both authors, BP and EH-L. EH-L drafted the manuscript, which was critically revised and finally approved by BP. Both authors agree to be accountable for all aspects of the work in ensuring that questions related to the accuracy or integrity of any part of the work are appropriately investigated and resolved.

### Conflict of interest statement

The authors declare that the research was conducted in the absence of any commercial or financial relationships that could be construed as a potential conflict of interest.
